# 2558. Association of Vancomycin-Induced Acute Kidney Injury with Trough Versus AUC Monitoring in Patients Receiving Extended Durations of Therapy

**DOI:** 10.1093/ofid/ofad500.2175

**Published:** 2023-11-27

**Authors:** C Tyler Pitcock, Katie L Wallace, Aric Schadler, David Burgess, Donna R Burgess, Sarah Cotner, Jeremy VanHoose, Eric Gregory

**Affiliations:** UK HealthCare, Lexington, Kentucky; University of Kentucky HealthCare, Lexington, KY; University of Kentucky Healthcare, Lexington, Kentucky; UK HealthCare, Lexington, Kentucky; UK HealthCare, Lexington, Kentucky; UK HealthCare, Lexington, Kentucky; UK HealthCare, Lexington, Kentucky; The University of Kansas Health System, Kansas City, MO

## Abstract

**Background:**

Vancomycin (VAN) therapy is associated with an increased risk of AKI. Previous studies suggest that AUC monitoring reduces the risk of AKI, but literature is lacking in patients receiving longer durations of VAN therapy. This study assessed the clinical impact of AUC monitoring on patients receiving at least 14 days of VAN.

**Methods:**

Adult inpatients at our academic medical center that received ≥ 14 days of VAN with levels obtained during a 4-year period were included in this analysis. Our primary outcome was the incidence of AKI between trough monitoring and AUC monitoring groups using the KDIGO change in serum creatinine from baseline criteria. Secondary outcomes included inpatient mortality, inpatient LOS, and ICU LOS. Multivariable logistic regression models were performed for AKI incidence and inpatient mortality.

**Results:**

A total of 582 patients were included. The majority of patients were male (58.2%) and Caucasian (94.3%). The median duration of VAN therapy was 23 days (IQR, 16-39). Patients within the trough monitoring group had a higher incidence of AKI compared to the AUC monitoring group (45.6% vs. 28.4%, p< 0.001). In each KDIGO stage, there was a higher incidence of AKI in the trough monitoring group compared to the AUC monitoring group (Stage 1: 22.0% vs. 15.9%, Stage 2: 10.7% vs. 7.6%, and Stage 3: 12.9% vs. 4.9%, p< 0.001). Logistic regression analysis showed AUC monitoring was associated with a 54% lower incidence of AKI (OR 0.46, 95% CI [0.31-0.69]). ICU LOS within the trough monitoring group was longer compared to the AUC monitoring group (13 days [6-24] vs. 10 days [3-19], p=0.008). All-cause inpatient mortality was numerically higher in the trough monitoring group (12.9% vs. 8.3%, p=0.078). In the second multivariable regression analysis, AUC monitoring was not a significant predictor of mortality, but patients with AKI were five times more likely to die (OR 5.00, 95% CI [2.40-10.46], p< 0.001).

**Conclusion:**

AUC monitoring can reduce the risk of AKI in patients receiving ≥ 14 days of VAN and indirectly reduce the risk of inpatient mortality. Consideration should be given to implementing AUC monitoring as a standard of practice.

**Disclosures:**

**All Authors**: No reported disclosures

